# Integrative Korean Medicine Treatment Including Ultrasound-Guided Shinbaro 2 Pharmacopuncture and Motion-Style Acupuncture Treatment for Acute Lumbar Disc Herniation with Foot Drop: A Case Report

**DOI:** 10.3390/healthcare14182949

**Published:** 2026-09-10

**Authors:** Young Suk Yoon, Jinyong Choi, Seok Yoon, Doori Kim

**Affiliations:** 1 Department of Korean Medicine Rehabilitation, Jamsil Jaseng Hospital of Korean Medicine, Seoul 05838, Republic of Korea; yoon406@gmail.com; 2 Jaseng Medical Academy, Jaseng Hospital of Korean Medicine, Seoul 06110, Republic of Korea; jinyong5440@jaseng.co.kr; 3 Graduate School of Transdisciplinary Health Sciences, Yonsei University, Seoul 03722, Republic of Korea; scotty@yuhs.ac; 4 Jaseng Spine and Joint Research Institute, Jaseng Medical Foundation, Seoul 06110, Republic of Korea

**Keywords:** pain relief, acute lumbar disc herniation, pharmacopuncture, motion-style acupuncture treatment, ultrasound-guided

## Abstract

**Highlights:**

**What are the main findings?**

- We described the clinical course of a patient with HIVD with radiculopathy and foot drop who underwent an integrative approach.
- Ultrasound-guided pharmacopuncture targeting the nerve root and Motion Style Acupuncture Technique (MSAT) for peripheral neuropathy may be considered as treatment options for patients with disc herniation.

**What are the implications of the main findings?**

- Ultrasound-guided pharmacopuncture may provide a feasible approach for targeting the affected nerve root in patients with acute disc herniation accompanied by radiculopathy.
- In patients with disc herniation and suspected peripheral neuropathy, double crush syndrome may be considered, and a multimodal treatment approach addressing both sites of nerve involvement may be beneficial.

**Abstract:**

**Background:** Herniated intervertebral disc (HIVD) with radiculopathy and foot drop can lead to substantial functional impairment. Although both pharmacopuncture and motion-style acupuncture treatment (MSAT) have demonstrated therapeutic potential individually, evidence on their combined use remains limited. In this single-case report, we described the clinical course of a patient with HIVD with radiculopathy and foot drop who underwent a novel approach combining ultrasound (US)-guided Shinbaro 2 pharmacopuncture targeting multiple nerve roots with tibialis anterior muscle motion-style acupuncture treatment (TA MSAT). **Case Presentation:** A 40-year-old man with lower back pain (LBP), radiculopathy, and foot drop attributed to acute HIVD was treated with high-dose US-guided Shinbaro 2 pharmacopuncture and TA MSAT. Outcomes were measured using the Numeric Rating Scale (NRS) for LBP, L5, and S1 radiculopathy; Manual Muscle Testing (MMT) for dorsiflexion and great toe extension; the Oswestry Disability Index (ODI) for lumbar function; the EuroQol Five-Dimension (EQ-5D) Index for quality of life; and the Patient Global Impression of Change (PGIC) for satisfaction. After 14 weeks of treatment, NRS scores for LBP, L5, and S1 radiculopathy decreased from 5 to 1, 8 to 1, and 8 to 1, respectively. MMT grades for dorsiflexion and great toe extension improved from 3 to 5 and 2 to 5, respectively, indicating full functional recovery. ODI and EQ-5D scores improved from 62.22 to 6.67 and 0.344 to 1.0, respectively. The PGIC score was 1, indicating clinical improvement. **Conclusions:** Our integrative approach involving US-guided pharmacopuncture and TA MSAT demonstrates therapeutic potential for the management of acute HIVD.

## 1. Introduction

A herniated intervertebral disc (HIVD), broadly defined as localized or focal displacement of the disc material beyond the boundaries of the intervertebral disc space [1], is most common in individuals aged 30–50 years, with a higher prevalence in men than in women [2]. HIVD is most frequently reported in the lumbar spine, with approximately 95% of disc herniations occurring at the L4–5 or L5–S1 levels [3]. In South Korea, a significant number of patients are diagnosed with intervertebral disc disorders (International Classification of Diseases, Tenth Revision, code M51). The socioeconomic burden of HIVD is substantial, with health insurance-covered medical expenses exceeding 600 million USD [4]. HIVD can be treated using both surgical and non-surgical approaches. Surgical interventions primarily include microdiscectomy, open discectomy, and microendoscopic discectomy [5]. Despite its minimally invasive nature, the use of microendoscopic surgery is limited by potential complications, including dural tears, postoperative hematoma, transient paresthesia, nerve root injury, and recurrence [6]. Furthermore, the prevalence of failed back surgery syndrome is steadily increasing, highlighting the importance of early screening and prevention [7].

Patients with HIVD often prioritize non-surgical options, including noninvasive interventions such as cognitive behavioral therapy (CBT), nonsteroidal anti-inflammatory drugs, and physical therapy. Other minimally invasive nonsurgical procedures, including steroid injection, radiofrequency ablation, and acupuncture, have also been recommended [5]. In 1951, South Korea officially recognized a dual healthcare system, licensing both Western medicine and Korean medicine (KM) [8]. KM, including acupuncture, pharmacopuncture, herbal medicine, and Chuna manual therapy, is a viable treatment option for patients with HIVD in South Korea. Notably, a 10-year analysis of Health Insurance Review and Assessment Service data indicated that KM was the primary treatment choice for a substantial number of patients with HIVD and lumbar spinal stenosis [9]. Furthermore, KM treatment as part of HIVD management demonstrated long-term therapeutic efficacy and sustainability [10].

Pharmacopuncture, which involves injection of therapeutic solutions into nerve roots affected by a herniated disc, is a KM modality utilized for pain management and the reduction in perineural inflammation [11]. Pharmacopuncture combines the pharmacological effects of herbal extracts with hydrodissection and other physical mechanisms [12]. The anti-inflammatory, neuroprotective, cartilage-regenerative, and analgesic effects of Shinbaro 2, one of the most widely used pharmacopuncture solutions for the treatment of HIVD [13], have been validated in preclinical studies [14]. Consequently, pharmacopuncture has received a Grade B recommendation (moderate-level evidence) from the Korean Clinical Practice Guidelines for HIVD [15].

In recent years, the clinical use of ultrasound (US)-guided pharmacopuncture, which integrates pharmacopuncture with real-time US imaging, has increased. US guidance enables the precise identification of neurovascular structures and the delivery of intensive treatment directly to the affected area [16], thereby enhancing procedural safety by avoiding vital neurovascular structures [17]. US guidance is essential for high-dose pharmacopuncture.

Motion-style acupuncture treatment (MSAT) is another therapeutic modality for various musculoskeletal disorders. Characterized by a combination of acupuncture and movement [18,19,20], it represents a distinct, novel approach compared with traditional acupuncture, which typically involves a static 15-min retention period. MSAT is primarily performed on the muscles and joints by inserting needles into the targeted muscle or joint, followed by passive movements applied by the physician [18].

Data on the individual therapeutic effects of US-guided high-dose pharmacopuncture and MSAT have been reported; however, reports on their combined application in patients with acute HIVD presenting with radiating pain and foot drop remain lacking.

Unlike conventional single lumbar nerve root blocks, this study applied US-guided pharmacopuncture to both the L5 and S1 nerve roots. Furthermore, to contribute to recovery from foot drop, TA MSAT was concurrently administered. In that we combined these modalities according to the patient’s multiple symptoms, this represents a novel therapeutic approach.

Given the retrospective nature of this single-patient case report, the requirement for ethical approval and informed consent was waived by the Institutional Review Board of Jaseng Hospital of Korean Medicine (JASENG 2026-03-003). The manuscript was prepared in accordance with the CARE guidelines for case reports.

## 2. Case Presentation

### 2.1. Clinical Features

A 40-year-old man with no history of lumbar disorders presented to the clinic on 29 June 2024, with chief complaints of left lower back pain (LBP), radiating pain in the left lower extremity, and left foot drop. His symptoms intensified 3 weeks before the initial visit, following >8 h of driving and 4 h of prolonged walking. He reported sharp, electrifying pain upon forward lumbar flexion, accompanied by left leg weakness and a dragging sensation during ambulation. Before the initial visit, the patient had received one medial branch block and one epidural steroid injection at another facility, with no significant clinical improvement.

### 2.2. Radiological Examination 

**Figure 1:**
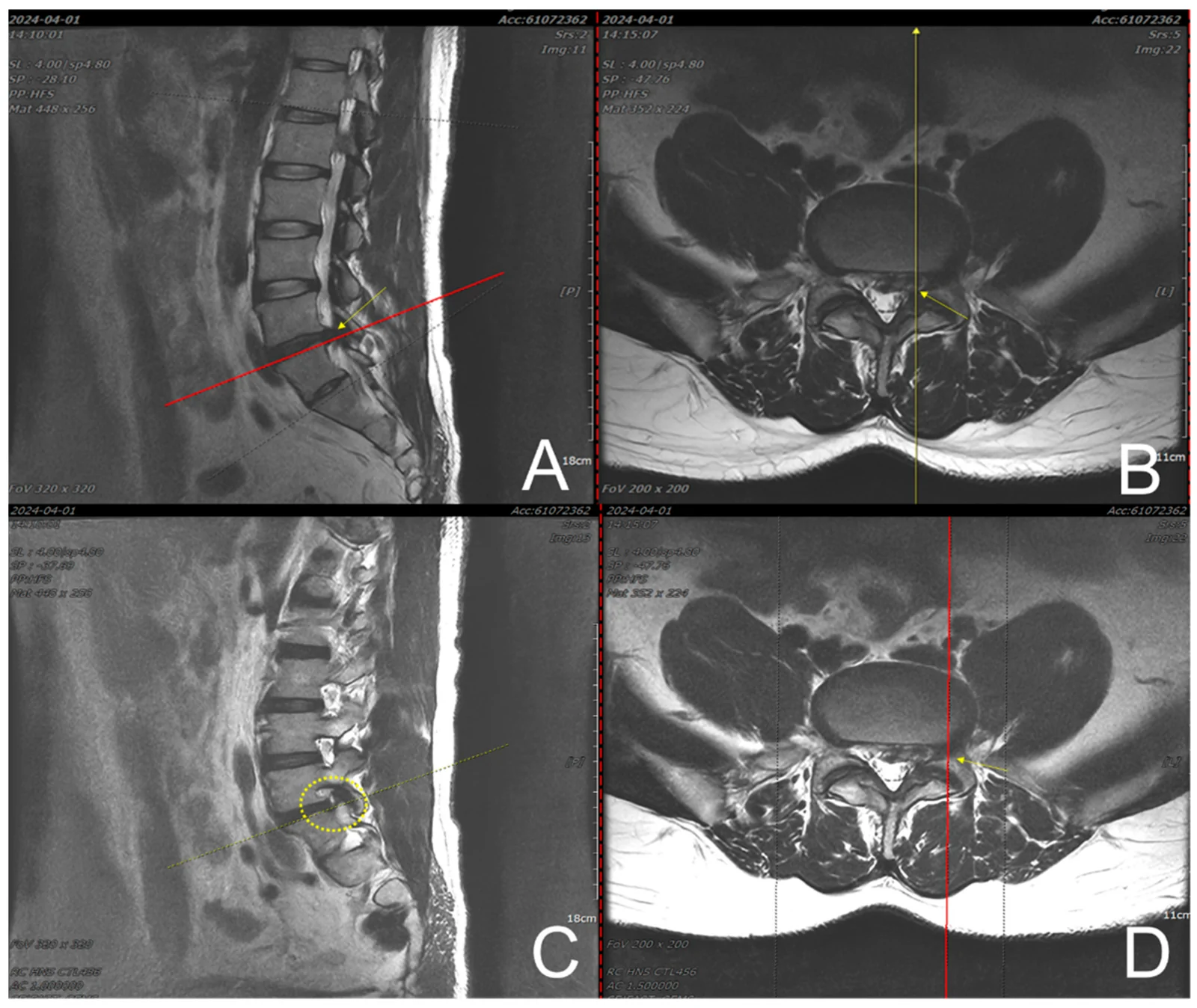
Lumbar spine MRI conducted on 1 April 2024. (**A**) T2-weighted turbo-spin-echo sagittal view. The yellow arrow indicates an extruded disc at the L5/S1 level. (**B**) T2-weighted turbo-spin-echo axial view. The yellow arrow indicates the compression of the left S1 traversing nerve root by a disc extrusion in the left paracentral zone at the L5/S1 level. (**C**) T2-weighted turbo-spin-echo sagittal view at the L5/S1 left extraforaminal zone. The yellow circle indicates a significantly compressed spinal canal compared with the upper levels. (**D**) T2-weighted turbo-spin-echo axial view at the L5/S1 left extraforaminal zone. The yellow arrow indicates the compression of the left L5 exiting nerve root by a disc extrusion in the left extraforaminal zone at the L5/S1 level. MRI, magnetic resonance imaging.

Magnetic resonance imaging scans of the lumbar spine obtained at an external clinic on 1 April 2024 revealed disc extrusion at the L5/S1 level on sagittal-view images. Axial images also showed an extrusion extending from the central to the left extraforaminal zone, resulting in compression of the left exiting L5 and transversing S1 nerve roots (Figure 1A,B). Significant compression of the exiting nerve roots was identified within the left extraforaminal zone at the L5/S1 level (Figure 1C,D). This finding was considered the primary cause of the patient’s foot drop. No additional MRI examination was performed during the treatment period.

### 2.3. Physical Examination (Rt./Lt.)

Physical examination findings were as follows. The range of motion was flexion 45°, extension 30°, lateral bending 30°/30°, and rotation 15°/15°. Special tests included a straight leg raise test of 60°/30° and a Patrick test of −/+, and deep tendon reflexes were within normal limits. Manual muscle test (MMT) grade (Right/Left) was dorsiflexion 5/3, great toe extension 5/2, and plantar flexion 5/5.Sensory examination revealed radiculopathic pain on the posterior aspect of the left leg and numbness on the lateral aspect of the left calf (L5 and S1 dermatomes). The L5 nerve root was compressed by an extraforaminal disc extrusion, resulting in reduced left dorsiflexion and great toe extension to grades 3 and 2, respectively.

### 2.4. Outcome Measures

LBP and radiating pain caused by L5 and S1 nerve root compression were assessed using the Numeric Rating Scale (NRS) at four specific time points: first visit, admission, discharge, and end of treatment. The patient’s quality of life and functional disability were evaluated using the EuroQol Five-Dimension (EQ-5D) index [21] and the Oswestry Disability Index (ODI) [22], respectively, at admission, discharge, and the end of treatment. Additionally, the Patient Global Impression of Change (PGIC) score was assessed at discharge. Motor strength for left-sided dorsiflexion and great toe extension was monitored daily throughout the treatment period using Manual Muscle Testing (MMT) grades [23].

### 2.5. Treatment

#### 2.5.1. Treatment Period

Following the initial visit on 29 June 2024, the patient underwent three outpatient treatment sessions. The patient was admitted for intensive treatment of foot drop on 26 July 2024 and remained hospitalized for 8 days until 2 August 2024. Following discharge, three additional outpatient follow-up sessions were conducted at 1-month intervals.

**Figure 2:**
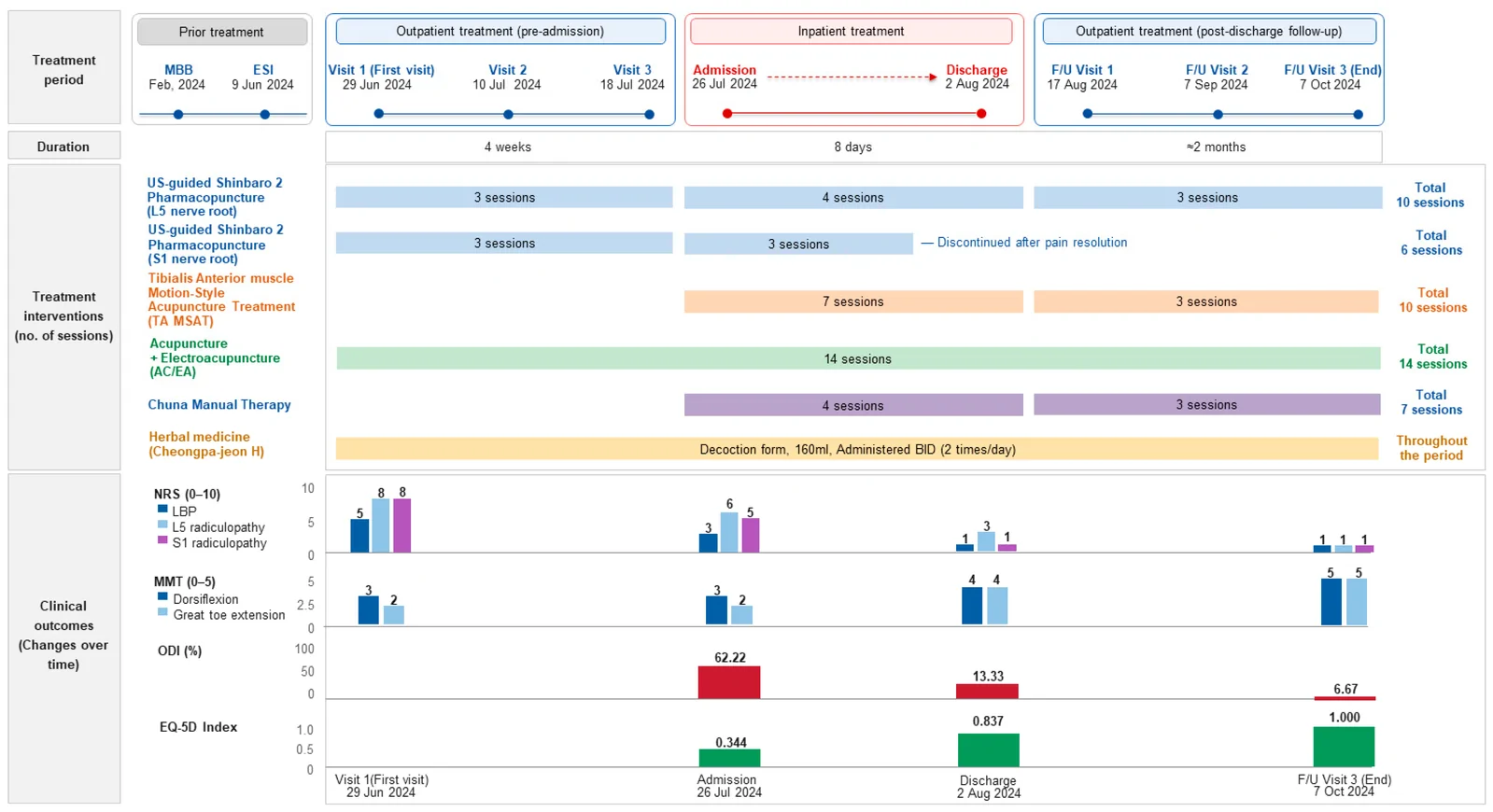
Treatment timeline and changes in clinical outcomes. MBB, medial branch block; ESI, epidural steroid injection; F/U, follow-up; US, ultrasound; TA MSAT, tibialis anterior muscle motion-style acupuncture treatment; AC, acupuncture; EA, electroacupuncture; LBP, lower back pain; NRS, numeric rating scale; MMT, manual muscle testing; ODI, Oswestry disability index; EQ-5D, EuroQol five-dimensions.

During this period, therapeutic interventions included US-guided pharmacopuncture for the L5 nerve root to alleviate nerve irritation, TA MSAT for muscle strength recovery, and concurrent acupuncture treatment to facilitate functional improvement. All interventions were performed by a board-certified specialist in Rehabilitation Medicine of Korean Medicine with more than 10 years of clinical experience. The treatment was completed on 7 October 2024 (Figure 2).

#### 2.5.2. US-Guided Pharmacopuncture

Ultrasound guidance was performed using a convex-array transducer (V8, Samsung Healthcare). US-guided pharmacopuncture was performed using 26-gauge (G) needles, targeting the L5 and S1 nerve roots. Considering the depth of the target nerve roots, a 90-mm needle (Jung Rim Medical Industrial, Jincheon, Republic of Korea) was used for the L5 nerve root, whereas a 60-mm needle (Jung Rim Medical Industrial, Jincheon, Republic of Korea) was used for the S1 nerve root. Shinbaro 2 (Jaseng Herbal Medicine Dispensary, Seongnam, Korea) was used as the pharmacopuncture solution. Shinbaro 2 pharmacopuncture solution is composed of nine herbs (Supplementary Materials S1) and is prepared at a Jaseng Herbal Medicine Dispensary accredited by the Korean Ministry of Health and Welfare, where the nine herbs are freeze-dried and subsequently diluted to a predetermined concentration.

To prevent infection, the physician wore sterile latex gloves, disinfected the patient’s skin with povidone–iodine solution, and maintained strict aseptic technique throughout the procedure. Before the procedure, the US probe was covered with a sterile surgical drape (Steri-Drape; 3M Health Care, Suzhou, China). Following the initial pre-scan, the entry point for the pharmacopuncture needle was marked with a surgical pen, and the area was thoroughly disinfected with povidone–iodine. To avoid radicular artery puncture, the injection was performed in power Doppler mode. Following the intervention, the entry point was re-disinfected, and a sterile adhesive bandage was applied.

#### 2.5.3. US-Guided S1 Nerve Root Pharmacopuncture

The patient complained of pain (pulling sensation) and numbness on the posterior aspect of the left thigh which were attributed to MRI-confirmed compression of the left S1 transversing nerve root. Under US guidance, 3 cc of Shinbaro 2 was injected targeting the S1 nerve root using the out-of-plane technique. Three sessions were conducted during the outpatient period, with an additional three sessions conducted during hospitalization. Following these interventions, the radiating pain completely resolved, and pharmacopuncture targeting the S1 nerve root was discontinued.

**Figure 3:**
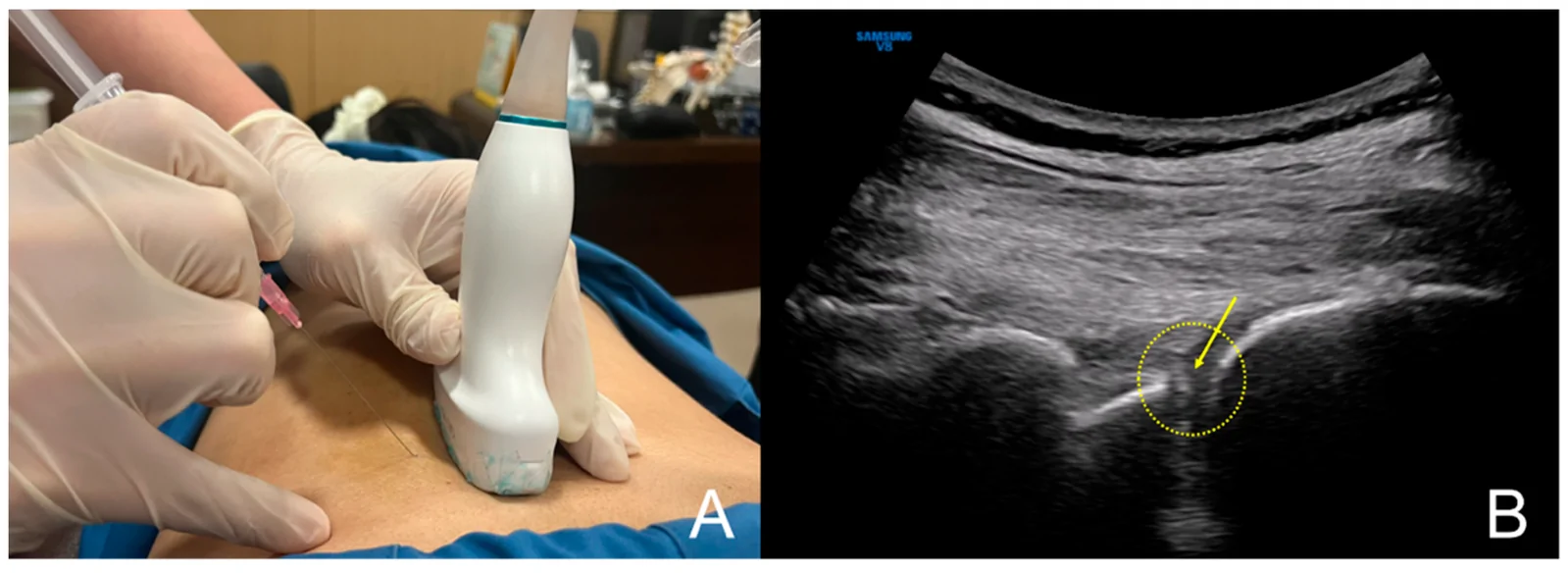
Ultrasound-guided S1 nerve root injection. (**A**) Out-of-plane approach for ultrasound-guided S1 nerve root pharmacopuncture. (**B**) The yellow circle indicates the S1 foramen, and the yellow arrow indicates the needle tip injecting the Shinbaro 2 pharmacopuncture solution.

The patient was placed in the prone position with a pillow under the abdomen, and the treatment bed was flexed to widen the interspinous space. In the longitudinal scan, the long axis of the sacrum was identified along with the facet joints of L5 and S1. The probe was moved caudally to identify the sacral S1 foramen. As the S1 nerve root passes beneath the S1 foramen, the needle tip was placed at the superior aspect of the foramen. Following confirmation of needle placement and the absence of intravascular injection via aspiration, 3 cc of Shinbaro 2 was injected, and its spread toward the foramen was confirmed (Figure 3).

#### 2.5.4. US-Guided High-Dose L5 Nerve Root Pharmacopuncture

The patient presented with radiating pain and foot drop symptoms which were attributed to MRI-confirmed compression of the left L5 exiting nerve root. Under US guidance, 6 cc of Shinbaro 2 was injected targeting the L5 nerve root using the in-plane technique. Ten sessions were conducted: three during the pre-hospitalization outpatient period, four during hospitalization, and three during the post-discharge outpatient period.

**Figure 4:**
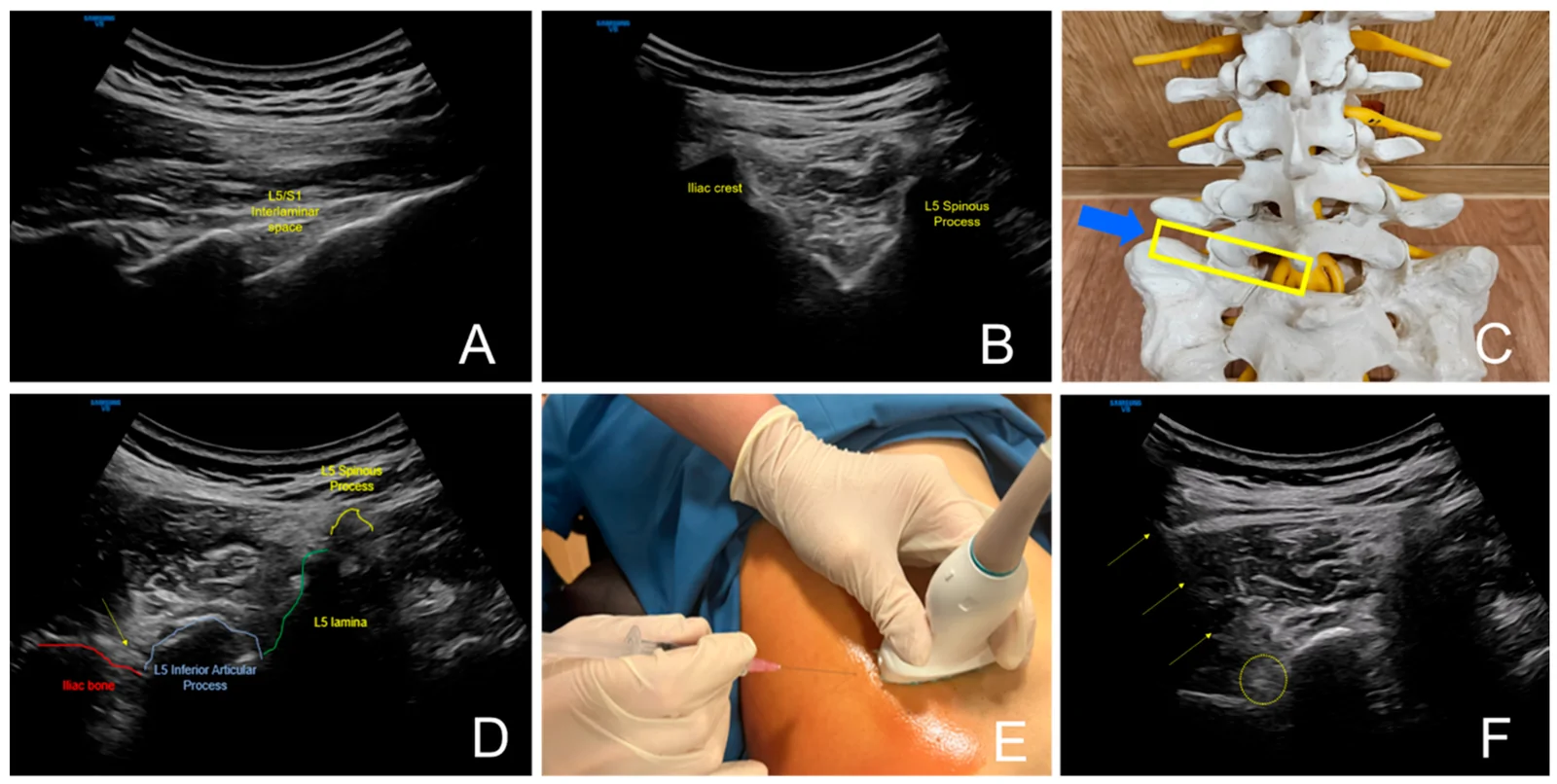
Ultrasound-guided L5 nerve root injection. (**A**) In the long-axis view, the interlaminar spaces of L4/5, L5/S1 are confirmed via the “horse heads sign.” (**B**) In the short-axis view, the L5 spinous process in the medial part and the iliac crest in the lateral part are confirmed. (**C**) Probe position and needle insertion direction for L5 nerve root pharmacopuncture injection. The yellow square indicates the probe location, and the blue arrow indicates the needle entry direction. (**D**) Applying clockwise rotation (using the pivot technique), the L5 spinous process (yellow line), L5 lamina (green line), L5 inferior articular process (blue line), and iliac bone (red line) are confirmed. The yellow arrow indicates the L5 nerve root. (**E**) The position of the probe and entry angle of the injection needle during the actual procedure. (**F**) The needle insertion path toward the L5 nerve root in the same plane as shown in C.

The patient was positioned in the same manner as for the S1 nerve root procedure. Following identification of the L5–S1 interlaminar space in the longitudinal scan (Figure 4A), the probe was rotated 90° to obtain a transverse view at the same level. In the transverse view, the lateral aspect of the image was initially obscured by the ilium (Figure 4B). Centered on the L5–S1 interlaminar space, a pivot technique was used to rotate the probe clockwise until the iliac crest was no longer visible laterally (Figure 4C). Once the L5 spinous process, lamina, inferior articular process, and iliac bone were clearly visualized, the posterior space between the lateral aspect of the L5 inferior articular process and iliac bone was identified as the exit point for the L5 nerve root (Figure 4D). After predicting the needle trajectory and the entry point, the needle was inserted using an in-plane approach with continuous monitoring of the needle tip (Figure 4E). Needle advancement was stopped when the needle tip reached the space posterior to the L5 inferior articular process. Following confirmation of needle placement and the absence of intravascular injection via aspiration, 6 cc of Shinbaro 2 was injected (Figure 4F).

Following the protocol of a previous study [24], the US-Guided pharmacopuncture was targeted at Kambin’s triangle, defined by the L5 exiting nerve root, the superior endplate of the S1 vertebra, and the anterior border of the S1 superior articular process. A 6 cc volume of Shinbaro 2 was administered to ensure sufficient diffusion of the solution throughout the peridiscal and perineural spaces within Kambin’s triangle. This high-volume approach was aimed at enhancing mechanical irrigation while leveraging the dose-dependent pharmacological effects of Shinbaro 2.

#### 2.5.5. MSAT

Despite three sessions of US-guided pharmacopuncture targeting the L5 nerve root, no improvement in foot drop symptoms was observed. Consequently, inpatient treatment was initiated for foot drop. Tibialis anterior muscle motion-style acupuncture treatment (TA MSAT) was performed 10 times for ten minutes per session: seven sessions during hospitalization and three during the post-discharge outpatient period.

**Figure 5:**
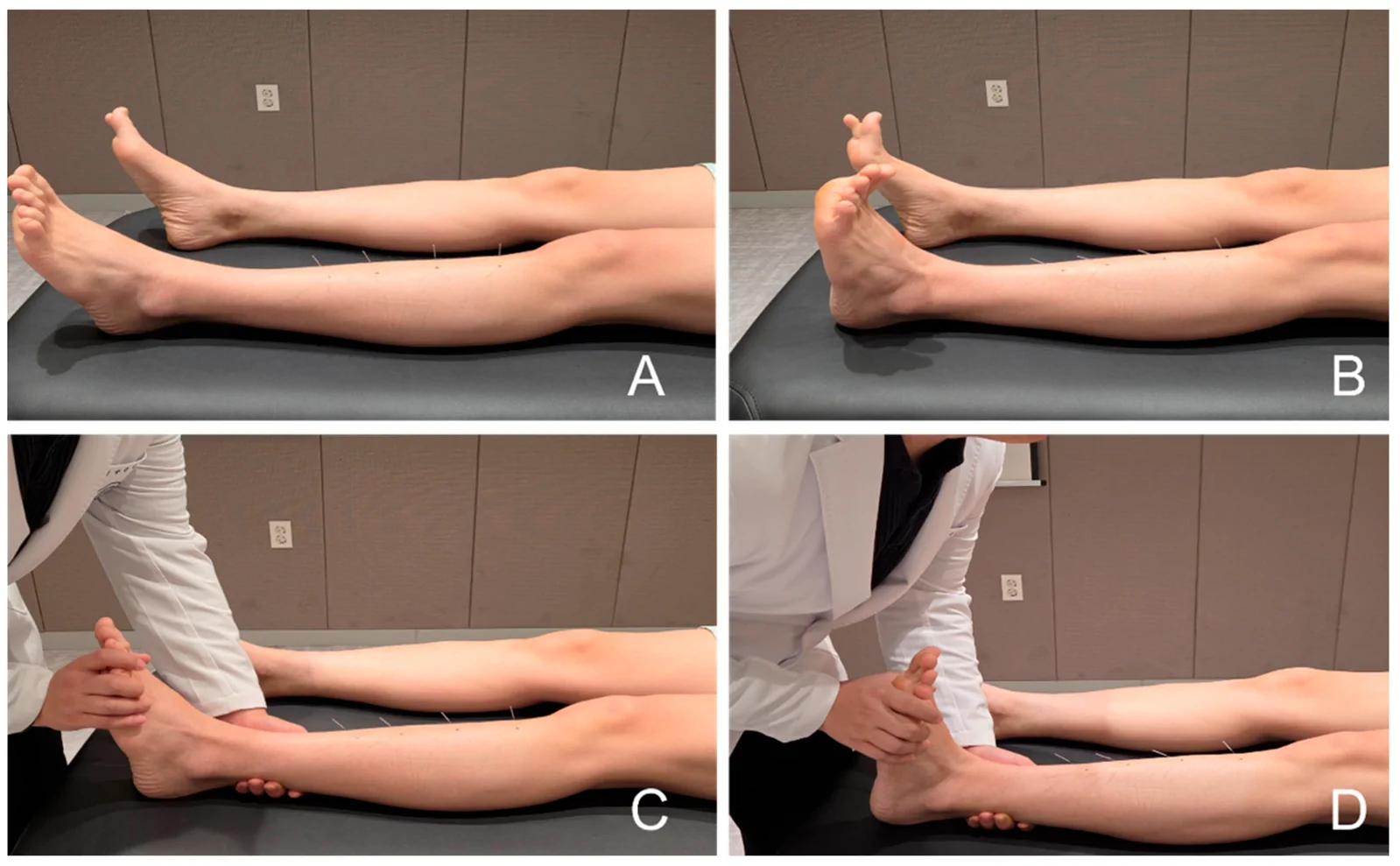
Tibialis anterior muscle MSAT. (**A**) Four acupuncture points needled for TA MSAT. (**B**) Active dorsiflexion performed by the patient during TA MSAT. (**C**) Preparatory posture for the physician to apply passive flexion during TA MSAT. (**D**) Physician applying passive flexion to the patient during TA MSAT.

The patient was positioned supine with both lower extremities extended to 180°. When four acupuncture needles (0.25 × 40-mm; Dongbang Medical, Seongnam, Republic of Korea) were inserted into the left tibialis anterior (TA) muscle, the medial border of the tibia was identified by palpation, and the needles were advanced medially to reach the TA muscle fibers. After insertion of four needles, the patient was instructed to perform two sets of 10 active ankle dorsiflexion repetitions, gradually increasing the range of flexion (Figure 5A,B). Subsequently, the physician held the patient’s ankle and performed two sets of 10 passive dorsiflexion repetitions to achieve maximum range (Figure 5C,D).

#### 2.5.6. Acupuncture and Electroacupuncture 

For acupuncture treatment, 0.25 × 40-mm needles (Dongbang Medical, Seongnam, Republic of Korea) were used to stimulate the bilateral BL23, BL24, BL25, BL26, GB30, and Huatuojiaji (EX-B2) points. To prevent atrophy of the TA muscle because of nerve entrapment, needles were inserted at left ST36, ST37, ST38, and ST39. Needles were retained for 15 min per session. Electroacupuncture was applied at a frequency of 2 Hz, with the intensity adjusted to the patient’s tolerance, for 15 min. Acupuncture and electroacupuncture were performed 14 times in total.

#### 2.5.7. Chuna Manual Therapy

Chuna manual therapy is a Korean medicine manual treatment classified into simple and complex techniques according to the Korean National Health Insurance scheme [25,26]. For the simple Chuna technique, the side-lying lumbar “pitch and roll” distraction method and the occipital correction technique were employed. For complex Chuna techniques, the prone both-pisiform lower thoracic flexion displacement correction, side-lying lumbar flexion displacement correction, and prone posteriorly rotated ilium correction/sacral lateral flexion correction techniques were used [27].

Chuna manual therapy was performed a total of 7 times, comprising 4 sessions during hospitalization and 3 sessions during the post-discharge outpatient period, with each session lasting approximately 10 min.

#### 2.5.8. Herbal Medicine

Cheongpa-jeon H, a prescription formulated by adding *Harpagophytum procumbens* to GCSB-5 [28], was administered as a decoction (160mL per dose) twice daily during treatment and continued after discharge. 

### 2.6. Changes in Outcome Measures

#### 2.6.1. NRS Score for LBP and Radiculopathy

Regarding LBP, the NRS score decreased from 5 at the initial visit to 1 at discharge. For S1 radiculopathy, the NRS score decreased from 8 to 1 during the same period. Similarly, for L5 radiculopathy, the NRS score decreased from 8 to 3 at discharge and was close to 1 by the end of treatment (Figure 2).

#### 2.6.2. MMT Grading for Dorsiflexion and Great Toe Extension

Dorsiflexion strength improved from grade 3 at the initial visit to grade 4 at discharge and reached grade 5 by the end of treatment. Similarly, great toe extension improved from grade 2 at the initial visit to grade 4 at discharge, reaching grade 5 by the end of treatment (Figure 2).

Despite four sessions of US-guided high-dose pharmacopuncture targeting the L5 nerve root, no improvement in muscle strength was observed. Consequently, the patient was hospitalized for concurrent TA MSAT. A total of 10 sessions of TA MSAT were performed, including 7 sessions during hospitalization and 3 sessions after discharge. At the end of treatment, dorsiflexion and great toe extension strength were observed to be grade 5.

#### 2.6.3. Sensory Deficits

Regarding sensory symptoms in the left lower extremity, radicular pain along the posterior aspect of the left leg and numbness along the lateral aspect of the left calf resolved by the end of treatment.

### 2.7. EQ-5D Index, ODI, and PGIC Scores

EQ-5D and ODI scores were measured three times: upon admission, at discharge, and at the end of treatment. The EQ-5D score increased from 0.344 at admission to 0.837 at discharge and finally reached 1.0 at the end of treatment (Figure 2). The ODI score improved from 62.22 at admission to 13.33 at discharge and further to 6.67 by the end of treatment (Figure 2). The PGIC score measured at discharge was 1, indicating significant improvement.

### 2.8. Adverse Events

The most frequently reported adverse effect was increased pain at the injection site, followed by increased radicular pain and lightheadedness [29]. Non-specific adverse events (e.g., vasovagal reactions/dizziness, localized bruising, hematoma, post-injection soreness, gastrointestinal discomfort) were systematically monitored, and no complications were reported. Specifically, during US-guided high-dose pharmacopuncture, strict safety protocols were implemented: prior to injection, blood aspiration was checked to prevent intravascular injection, the injection flow rate was carefully controlled, procedure-induced nerve irritation was monitored, and 2 h of post-injection bed rest was prescribed. No serious adverse events, including infection or vascular complications, occurred. Laboratory tests performed at admission and discharge revealed no abnormal findings.

## 3. Discussion

We described the clinical course of a patient with HIVD with radiculopathy and foot drop who underwent integrative approach involving US-guided Shinbaro 2 pharmacopuncture targeting multiple nerve roots with TA MSAT. 

Corticosteroid injection is the most widely used minimally invasive procedure for these conditions. Steroids mitigate inflammation around nerves by suppressing edema and inhibiting inflammatory mediators [30]. However, because of their immunosuppressive effects, injections prior to joint replacement or spinal surgery have been linked to increased postoperative infection rates. A minimum interval of 3 months between steroid injection and surgery is recommended to minimize the risk of infection [31,32]. Furthermore, corticosteroids dysregulate glucose metabolism, leading to steroid-induced diabetes [33].

Pharmacopuncture using herbal extracts is not associated with immunosuppression. Kim et al. [34] investigated the side effects of pharmacopuncture in 80,523 patients and reported that most cases were associated with bee venom, whereas infection-related complications accounted for only 0.002%.

The Shinbaro 2 pharmacopuncture used in this study is one of the most widely investigated agents for spinal treatment [24,35]. In animal studies, Shinbaro 2 pharmacopuncture has demonstrated anti-inflammatory and disc-protective effects in rat models of HIVD and stenosis [14,36].

In this case, the medial branch may not have been the appropriate treatment target, whereas the epidural steroid injection may have provided primarily transient pain relief, with its effects not being sustained.

In our case, high-dose Shinbaro 2 pharmacopuncture targeting the L5 nerve root was performed under US guidance to maximize both chemical mechanisms and mechanical effects, including hydrodissection. US guidance enhances the safety and efficacy of pharmacopuncture [37,38]. In the present case, US-guided pharmacopuncture may have contributed to improvement of radiating pain attributed to L5 and S1 nerve root compression. However, given the relatively slower improvement in foot drop, intensive inpatient treatment and TA MSAT were additionally applied. To date, only two studies have examined the use of US-guided Shinbaro 2 pharmacopuncture targeting the L4 nerve root and the common peroneal nerve for patients with lumbar HIVD [24,38]. Given the anatomical location of the L5 nerve root, various approaches are available for US-guided injections. The L5 nerve root lies posterior to the L5–S1 facet joint; however, the ilium often hinders access with an in-plane technique. Although Kim et al. [39] used an out-of-plane injection technique into the inter-transverse space, we employed an in-plane technique to continuously track the needle path throughout the procedure, consistent with prior methods [40,41].

In the US-guided S1 nerve approach, pharmacopuncture was performed superior to the S1 foramen. Given the surrounding bony structures, the out-of-plane approach is considered the most feasible [42]. For S1 nerve root injection, the needle tip was not advanced into the S1 foramen, as no significant clinical differences have been reported between the perineural and paraneural approaches [43].

Foot drop can be caused by L5 nerve root compression or deep peroneal nerve (DPN) entrapment. The DPN primarily comprises motor fibers that innervate all ankle and toe extensors [44]. Consequently, DPN entrapment leads to lower limb muscle weakness or foot drop.

In the present case, foot drop showed minimal improvement after high-dose pharmacopuncture targeting the L5 nerve root. This suggests that the patient’s foot drop was more likely to be associated with a peripheral neuropathy. The diagnosis of peroneal neuropathy is generally based on the patient’s medical history and physical examination [45]. In this case, a Tinel’s sign at the peroneal tunnel was assessed after admission to evaluate the possibility of peroneal neuropathy, and a positive finding was observed. In addition, there was no history of trauma, and no structural abnormalities, such as osteophytes, were identified on the left foot X-ray. Nerve conduction studies of the peroneal nerve and assessment of transient improvement following nerve block were not performed.

Double crush syndrome (DCS) consists of impingement of the spinal and peripheral nerves [46]. The mechanism of DCS is that a single lesion in the proximal portion of a nerve predisposes that nerve to a second lesion distally, especially when the nerve passes through a narrow anatomical canal [47]. Maejima et al. [48] performed surgery to treat L5 radiculopathy in a patient with lumbar disc herniation. Although the patient’s upper-leg symptoms improved after surgery, the symptoms in the lower leg did not improve. During follow-up, the patient was diagnosed with peroneal neuropathy and underwent decompression surgery, after which the lower-leg symptoms improved.

This case was considered to represent DCS of the lower limb, presenting with L5 radiculopathy and peroneal neuropathy. Similar to the case reported by Maejima et al. [48], peroneal neuropathy was considered following minimal improvement despite intervention targeting the L5 nerve root. Accordingly, TA MSAT was applied to address the suspected peroneal neuropathy. In DCS, symptoms from one lesion may mask those from another, and Maejima et al. [48] suggested that L5 nerve dysfunction may mask symptoms caused by peroneal nerve compression. DCS is known to be challenging to diagnose because of this complex combination of nerve lesions.

In the therapeutic framework for patients presenting with foot drop, the first step is to understand the underlying etiology. As demonstrated in this study, L5 nerve root compression secondary to lumbar HIVD represents the most common etiology, while secondary complications in stroke patients and peroneal nerve neuropathy also constitute major causative factors. 

According to the current German Society of Neurology (Deutsche Gesellschaft für Neurologie, DGN) guideline on lumbar radiculopathy [49,50], a muscle grading of >3/5 constitutes a relative indication for surgery, whereas a grading of ≤3/5 serves as an absolute indication. In a study by Telfeian et al., five patients presenting with foot drop due to Lumbar HIVD underwent transforaminal endoscopic decompression, with a mean preoperative tibialis anterior muscle strength of 2.6/5 [51].

Conventional approaches for treating this condition include activity modification, bracing, and physical therapy [52], as well as electrical stimulation of weakened muscles [52]. Additionally, Waseem et al. [53] proposed a surgical option for foot drop. 

Clinical data defining the precise timing of surgery for decompression of the peroneal nerve, which directly causes foot drop, remain limited. As long as the neuronal cell body remains intact, axonal sprouting occurs for up to six months following injury; however, regenerative capacity begins to decline after three months due to various structural and molecular changes in the distal stump [54].

To address this refractory symptom, TA MSAT was concurrently applied to alleviate foot drop. Lee et al. [55,56] successfully treated foot drop using TA MSAT in patients with lumbar HIVD; however, the underlying therapeutic mechanism was not elucidated. The authors propose the following mechanism. The acupoints used in TA MSAT (ST36, ST37, ST38, and ST39) are located within the TA muscle, a representative muscle of the ankle extensor. Given that the DPN consistently courses between the TA and extensor hallucis longus muscles [57], mechanical stimulation from needles inserted to a depth of approximately 4 cm, combined with passive and active ankle movements, may help resolve the entrapment. Although needle insertion into muscle fibers has been reported to enhance cutaneous and muscular blood flow [58], the integration of active movement into MSAT may potentially contribute to this effect.

The possibility of spontaneous improvement in acute lumbar disc herniation should be taken into account when interpreting the findings of the present case. Spontaneous regression of herniated disc material has been reported in previous studies; Chiu et al. reported that spontaneous regression occurred in 70% of extruded discs managed conservatively [59]. Accordingly, a substantial degree of spontaneous regression may also have occurred during the 14-week observation period in the present case. Nevertheless, the temporal sequence in which dorsiflexion strength remained at MMT grade 3 and first improved to grade 4 after two sessions of TA MSAT, reaching grade 5 after eight sessions, suggests a possible contribution of the additional treatment. 

This case study had some limitations. First, it involved a report of a single case. Second, this study did not involve comparisons between pharmacopuncture and other injectable therapies or between conventional acupuncture and MSAT. Cautious, exploratory observational studies or prospective registry-based case series are warranted to provide higher-level evidence. Third, because the patient received multiple interventions, it is impossible to determine which specific treatment contributed to the observed recovery. Fourth, follow-up MRI was not performed to assess the extruded disc after completion of treatment.

## 4. Conclusions

A patient with acute HIVD accompanied by radiculopathy and foot drop underwent a 14-week integrative medicine approach. During the treatment period, improvements were observed in LBP, L5 and S1 radiculopathy, and foot drop, along with changes in EQ-5D and ODI scores. A key strength of this report is its novel integrative approach combining US-guided pharmacopuncture with TA MSAT, which may offer potential benefits in terms of the safety and treatment process for acute HIVD accompanied by foot drop.

## Data Availability

All data generated or analyzed during this study are included in this published article. If additional information is required, further details may be shared upon reasonable request to the corresponding author. However, access to certain data may be restricted because of privacy and confidentiality considerations, and not all requested data can be made publicly available.

## Supplementary Materials

The following supporting information can be downloaded at: https://www.mdpi.com/article/10.3390/healthcare14182949/s1. Supplementary Materials S1: Composition of Shinbaro 2 Pharmacopuncture.

## Abbreviations

The following abbreviations are used in this manuscript:

HIVD	Herniated intervertebral disc
MSAT	Motion-style acupuncture treatment
TA	Tibialis anterior
TA MSAT	Tibialis anterior muscle motion-style acupuncture treatment
LBP	Lower back pain
NRS	Numeric Rating Scale
MMT	Manual Muscle Testing
MRI	Magnetic resonance imaging
ODI	Oswestry Disability Index
EQ-5D	EuroQol Five-Dimensions
PGIC	Patient Global Impression of Change
CBT	Cognitive behavioral therapy
KM	Korean medicine
US	Ultrasound
DPN	Deep peroneal nerve
DCS	Double Crush Syndrome
