# Supplementary material for: Integrative Korean Medicine Treatment Including Ultrasound-Guided Shinbaro 2 Pharmacopuncture and Motion-Style Acupuncture Treatment for Acute Lumbar Disc Herniation with Foot Drop: A Case Report
Source: Healthcare (Basel). 2026 Sep 10;14(18):2949. doi: 10.3390/healthcare14182949 (PMC13606044; doi:10.3390/healthcare14182949)
Supplement: Supplementary file 1 [file healthcare-14-02949-s001.zip › healthcare-4479641-supplementary.pdf]

Supplementary Materials S1. Composition of Shinbaro 2 Pharmacopuncture

Rhizomes of Cibotium barometz (0.0013 g/mL), roots of Saposhnikovia divaricata (0.0013), stem barks of Eucommia ulmoides (0.0013), stems and roots of Acanthopanax sessiliflorus (0.0013), rhizomes and roots of Ostericum koreanum (0.0013), roots of Angelica pubescens (0.0013), roots of Achyranthes japonica (0.0013), Scolopendra subspinipes (0.0013), roots of Paeonia albiflora (0.0027)
